# Immune and environment‐driven gene expression during invasion: An eco‐immunological application of RNA‐Seq

**DOI:** 10.1002/ece3.5249

**Published:** 2019-05-09

**Authors:** Daniel Selechnik, Mark F. Richardson, Richard Shine, Gregory P. Brown, Lee Ann Rollins

**Affiliations:** ^1^ School of Life and Environmental Sciences (SOLES) University of Sydney Sydney New South Wales Australia; ^2^ Deakin Genomics Centre, School of Life and Environmental Sciences Deakin University Geelong Victoria Australia; ^3^ Centre for Integrative Ecology, School of Life and Environmental Sciences Deakin University Geelong Victoria Australia; ^4^ Evolution & Ecology Research Centre, School of Biological, Earth and Environmental Sciences UNSW Sydney Sydney New South Wales Australia

**Keywords:** *Bufo marinus*, cane toad, compositional data analysis, enemy release hypothesis, invasive species, spatial sorting

## Abstract

Host–pathogen associations change rapidly during a biological invasion and are predicted to impose strong selection on immune function. It has been proposed that the invader may experience an abrupt reduction in pathogen‐mediated selection (“enemy release”), thereby favoring decreased investment into “costly” immune responses. Across plants and animals, there is mixed support for this prediction. Pathogens are not the only form of selection imposed on invaders; differences in abiotic environmental conditions between native and introduced ranges are also expected to drive rapid evolution. Here, we use RNA‐Seq to assess the expression patterns of immune and environmentally associated genes in the cane toad (*Rhinella marina*) across its invasive Australian range. Transcripts encoding mediators of costly immune responses (inflammation, cytotoxicity) showed a curvilinear relationship with invasion history, with highest expression in toads from oldest and newest colonized areas. This pattern is surprising given theoretical expectations of density dynamics in invasive species and may be because density influences both intraspecific competition and parasite transmission, generating conflicting effects on the strength of immune responses. Alternatively, this expression pattern may be the result of other evolutionary forces, such as spatial sorting and genetic drift, working simultaneously with natural selection. Our findings do not support predictions about immune function based on the enemy release hypothesis and suggest instead that the effects of enemy release are difficult to isolate in wild populations, especially in the absence of information regarding parasite and pathogen infection. Additionally, expression patterns of genes underlying putatively environmentally associated traits are consistent with previous genetic studies, providing further support that Australian cane toads have adapted to novel abiotic challenges.

## 1 INTRODUCTION

Invasive species pose a massive threat to biodiversity (Bax, Williamson, Aguero, Gonzalez, & Geeves, 2003; Clavero, Brotonsa, Pons, & Sol, 2009). The potential for pathogens to limit the impact of invaders, or to exacerbate that impact, makes it critical to understand how the process of range expansion alters invader immunity. In equilibrial systems, pathogen‐mediated selection (PMS) favors host individuals with traits that enable them to resist or tolerate infections (Spurgin & Richardson, 2010), but it is unclear whether the same traits remain favorable during invasion. Furthermore, some invasive traits follow patterns that are not explained by selection (Berthouly‐Salazar, Rensburg, Roux, Vuuren, & Hui, 2012; Lowe, Muhlfeld, & Allendorf, 2015). This may be because the dispersive tendencies of invaders give rise to additional evolutionary forces: (a) As an invasive population expands, genetic drift may reduce genetic diversity across the range (Rollins, Woolnough, Wilton, Sinclair, & Sherwin, 2009) and modify phenotypic traits. (b) An expanding invasion front is dominated by individuals with the highest rates of dispersal simply because the fastest arrive at new areas first and can only breed with each other (spatial sorting; Shine, Brown, & Phillips, 2011). Thus, a geographic separation of phenotypes occurs; traits that enhance individual fitness are favored in established populations, and traits that enhance dispersal rate are common in expanding populations (Hudson, Brown, & Shine, 2016; Shine et al., 2011). (c) Admixture between individuals from different introductions or sources, as well as hybridization, may also drive evolutionary change in some invasive populations (Mader, Castro, Bonatto, & Freitas, 2016). Alternatively, some traits differ simply due to heightened environmental variability experienced by invaders and do not reflect evolutionary change. Given the complex possibilities of adaptive and nonadaptive changes imposed on immune systems during range expansion, identifying their underlying basis is an important task.

**Figure 1:**
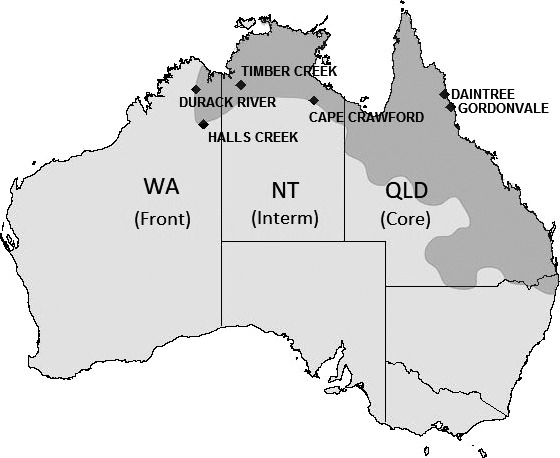
Geographic distribution of the cane toad in Australia (dark gray region). Since arriving in Queensland in 1935, cane toads have further expanded their range through New South Wales, the Northern Territory, and into Western Australia. Black diamonds indicate our toad collection sites (from east to west): QLD (Gordonvale and Daintree, *N* = 5 each), NT (Cape Crawford and Timber Creek, *N* = 4 each), and WA (Caroline Pool and Durack River, *N* = 5 each). Map adapted from Tingley, et al. (2017)

We examined the effects of range expansion on expression of immune and environmentally associated genes in the invasive Australian cane toad (*Rhinella marina*) using RNA‐Seq data from whole spleen tissue from individuals collected from long‐established areas in Queensland (QLD, the “range core”), geographically “intermediate” areas in the Northern Territory (NT), and the leading edge of the range expansion in Western Australia (WA, the “invasion front”; Figure 1). The invasive range of cane toads in Australia includes highly varied environments; climatic conditions in the range core are similar to those in the native range (Central and South America), but intermediate areas and the invasion front receive much less annual rainfall (2,000–3,000 mm in QLD, 400–1,000 mm in NT and WA) and have higher annual mean temperatures (21–24°C in QLD, 24–27°C in NT and WA; Bureau of Meteorology A.G., 2018). Toads cluster genetically based on these environmental patterns: Toads from the range core are genetically distinct from those from intermediate areas and the invasion front (Selechnik, Richardson, & Shine, 2019). Furthermore, loci putatively under selection are involved in tolerance of temperature extremes and dehydration (Selechnik et al., 2019). Traits such as locomotor performance at high temperatures also follow this pattern (Kosmala, Brown, Christian, Hudson, & Shine, 2018), but others do not. For example, behavioral propensity for exploration increases with distance from the introduction site (Gruber, Brown, Whiting, & Shine, 2017). Traits such as leg length (Hudson et al., 2016), spleen size, and fat body mass (Brown, Kelehear, Shilton, Phillips, & Shine, 2015b) follow a U‐shaped (curvilinear) pattern across the range, in which they are smallest in toads from intermediate areas of the range and larger at either end.

One process likely to be important in shaping immune systems during range expansion is loss of parasites and pathogens. The enemy release hypothesis (ERH) predicts that the processes of introduction and range expansion decrease rates of infections with coevolved pathogens and parasites in invasive hosts due to the former's inability to spread effectively and persist in novel environmental conditions (Colautti, Ricciardi, Grigorovich, & MacIsaac, 2004). Because of this, Lee and Klasing (2004) predict that invaders may downregulate powerful immune responses such as systemic inflammation due to a decreased need (Cornet, Brouat, Diagne, & Charbonnel, 2016; Lee & Klasing, 2004; Martin, Hopkins, Mydlarz, & Rohr, 2010). Such immune responses are also costly due to energetic expenditure (the reduction of nutrients available for partitioning across tissues due to their use in mounting immune responses; Klasing & Leshchinsky, 1999) and to the potential for collateral damage (tissue injury due to the effects of the immune response; Martin et al., 2010). Reduced energetic investment into these immune responses may enhance invasion success (McKean & Lazzaro, 2011). Nonetheless, loss of immunocompetence (the ability to mount a normal immune response after exposure to an antigen; Janeway, Travers, & Walport, 2001) could render invaders susceptible to infection by novel pathogens and parasites in their introduced range (Cornet et al., 2016; Lee & Klasing, 2004). Thus, invaders are predicted to exhibit lower investment in costly (but not all) immune responses than are seen in their native ranges (Cornet et al., 2016; Lee & Klasing, 2004). Enemy release is likely to be relevant to the ecoimmunology of cane toads in Australia.

Consistent with the ERH, many species of bacteria, protozoan, and metazoan parasites of cane toads have been left behind in the native range (Selechnik, Rollins, Brown, Kelehear, & Shine, 2017a). A major parasite (lungworm *Rhabdias pseudosphaerocephala*) from the native range that infects toad populations in the Australian range core is absent from toads at the invasion front (Phillips et al., 2010); this lungworm has been reported to increase mortality by 17% in metamorph cane toads (Kelehear, Webb, & Shine, 2009) and 6% in adult cane toads (Finnerty, Shine, & Brown, 2017). Conversely, the Australian soil bacterium *Brucella* (*Ochrobactrum*) *anthropi* causes spinal spondylosis in toads primarily at the invasion front (Brown, Shilton, Phillips, & Shine, 2007), which may represent a novel infection that forces invaders to remain immunocompetent. Furthermore, Rhimavirus A has only been detected in transcriptomes of toads from areas relatively close to the invasion front (Russo et al., 2018). Invasion history has complex effects on toad immunity (Brown, Phillips, Dubey, & Shine, 2015c; Brown & Shine, 2014; Selechnik, West, et al., 2017b).

The loss of pathogens underlying the ERH depends on a decline in pathogen transmission, which likely occurs when host densities are lower. The densities of many invasive populations follow a “traveling wave,” in which population density is low at recently colonized areas (e.g., the invasion front), high in areas that have been colonized for several years (e.g., intermediate areas), and low at long‐colonized areas (e.g., the range core; Hilker, Lewis, Seno, Langlais, & Malchow, 2005; Simberloff & Gibbons, 2004). Although absolute population densities of cane toads across Australia are unknown, toads appear to follow this trend as well (Brown, Kelehear, & Shine, 2013; Freeland, Delvinquier, & Bonnin, 1986), as does at least one of their major parasites (*Rhabdias pseudosphaerocephala*). This parasitic lungworm is absent from toads at the invasion front and is most prevalent in toads from intermediate areas (Brown, Kelehear, et al., 2015b; Phillips et al., 2010). Therefore, we predicted that the expression of immune genes may follow a curvilinear pattern, in which those encoding mediators of costly immune responses (e.g., inflammation) may be more highly expressed in toads from intermediate areas than in toads from the core or front. In terms of environmentally associated genes, we predicted that expression patterns would depend on the functional roles of the genes; for example, genes involved in aridity tolerance may be differentially expressed between toads from the (moist) range core and toads throughout the rest of the (more arid) range.

## 2 MATERIALS AND METHODS

### 2.1 Sample collection and RNA extraction

In April and May of 2014 and 2015, we collected adult female cane toads from six locations along an invasion transect (Figure 1): Gordonvale, QLD (*N* = 5, range core, 17.0972S 145.7792E); Daintree, QLD (*N* = 5, range core, 16.25S 145.3167E); Cape Crawford, NT (*N* = 4, intermediate, 16.6667S 135.8E); Timber Creek, NT (*N* = 4, intermediate, 15.6453S 130.4744E); Halls Creek, WA (*N* = 5, invasion front, 18.2265S 127.759E); and Durack River, WA (*N* = 5, invasion front, 15.9419S 127.2202E). We euthanized toads using 150 mg/kg sodium pentobarbital, decapitated them as soon as they became unresponsive, and excised their spleens immediately following decapitation. We selected spleen tissue for our investigation because mature immune cells travel to secondary lymphoid tissue (spleen, lymph nodes, and mucosa‐associated lymphoid tissue) for activation through pathogenic encounter (Janeway et al., 2001). Thus, the cellular compositions of these tissues should reflect the host's immune functioning. Each spleen was initially preserved in RNAlater (Qiagen), kept at 4°C for <1 week, and then drained and transferred to a −80°C freezer for long‐term storage.

Prior to RNA extraction, we flash‐froze all spleens individually in liquid nitrogen and ground them with a mortar and pestle to lyse preserved tissue. We carried out RNA extractions using the RNeasy Lipid Tissue Mini Kit (QIAGEN) following the manufacturer's instructions, with an additional genomic DNA removal step using on‐column RNase‐free DNase treatment (Qiagen). We quantified the total RNA extracted using a Qubit RNA HS assay on a Qubit 3.0 fluorometer (Life Technologies). Extracts were then stored at −80°C until sequencing was performed.

### 2.2 Sequencing

Prior to sequencing, we added 4 µl of either mix 1 or mix 2 of External RNA Controls Consortium (ERCC; Thermo Fisher Science) spike‐in solutions diluted 1:100 to 2 µg of RNA to examine the technical performance of sequencing (Table S1). Macrogen (Macrogen Inc., ROK) constructed mRNA libraries using the TruSeq mRNA v2 sample kit (Illumina Inc.), which included a 300 bp selection step. All samples from the core and the front were individually barcoded and sequenced across two lanes of Illumina HiSeq 2,500 (Illumina Inc.); samples from intermediate areas were sequenced in a separate batch on a single lane of Illumina HiSeq 2500 (also individually bardcoded). Capture of mRNA was performed using the oligo dT method, and size selection parameter choices were made according to the HiSeq2500 manufacturer's protocol. Overall, this generated 678 million paired‐end 2 × 125 bp reads. Raw sequence reads are available as FASTQ files in the NCBI short read archive (SRA) under the BioProject Accession PRJNA395127.

### 2.3 Data preprocessing, alignment, and expression quantification

First, we examined per base raw sequence read quality (Phred scores) and GC content, and checked for the presence of adapter sequences for each sample using FastQC v0.11.5 (Andrews, 2010). We then processed raw reads (FASTQ files) from each sample with Trimmomatic v0.35 (Bolger, Lohse, & Usadel, 2014), using the following parameters: ILLUMINACLIP:TruSeq3‐PE.fa:2:30:10:4 SLIDINGWINDOW:5:20 AVGQUAL:20 MINLEN:36. This removed any adaptor sequences, trimmed any set of 5 contiguous bases with an average Phred score below 20 and removed any read with an average Phred score below 20 or sequence length below 36 bp.

As a reference for alignment, we used the annotated *R. marina* transcriptome (Richardson et al., 2018), which was constructed from brain, spleen, muscle, liver, ovary, testes, and tadpole tissue. We conducted per sample alignments of our trimmed FASTQ files to this reference using STAR v2.5.0a (Patro, Duggal, Love, Irizarry, & Kingsford, 2017) in basic two‐pass mode with default parameters, a runRNGseed of 777, and specifying BAM alignment outputs. We used the BAM outputs to quantify transcript expression using Salmon v0.8.1 (Patro et al., 2017) in alignment mode with libtype = IU, thus producing count files.

### 2.4 Count filtering and log‐ratio transformations

Most methods for analyzing RNA‐Seq expression data assume that raw read counts represent absolute abundances (Quinn, Richardson, Lovell, & Crowley, 2017). However, RNA‐Seq count data are not absolute and instead represent relative abundances as a type of compositional count data (Quinn, Erb, Richardson, & Crowley, 2018c; Quinn, Richardson, et al., 2017). Using methods that assume absolute values is invalid for compositional data (without first including a transformation) because the total number of reads (library size) generated from each sample varies based on factors such as sequencing performance, making comparisons of the actual count values between samples difficult (Fernandes et al., 2014; Quinn, Erb, et al., 2018c). As such, relationships within RNA‐Seq count data make more sense as ratios, either when compared to a reference or to another feature within the dataset. Hence, we analyzed our count data (from Salmon) taking the compositional nature into account using the log‐ratio transformation (Aitchison & Egozcue, 2005; Erb & Notredame, 2016; Lovell, Pawlowsky‐Glahn, Egozcue, Marguerat, & Bahler, 2015; Quinn, Erb, et al., 2018b; Quinn, Richardson, et al., 2017). Our total number of expressed transcripts across all toads was 22,930. To filter out transcripts with low expression, we removed transcripts that did not have at least 10 counts in 10 samples. This reduced our list of expressed transcripts to 18,945. We then used the R (Team, 2016) package ALDEx2 v1.6.0 (Fernandes, Macklaim, Linn, Reid, & Gloor, 2013) to perform an interquantile log‐ratio (iqlr) transformation of the transcripts’ counts as the denominator for the geometric mean calculation (rather than centered log‐ratio transformation) because it removes the bias of transcripts with very high and low expression that may skew the geometric mean (Quinn, Richardson, et al., 2017). To circumvent issues associated with other normalization methods, we used ALDEx2 to model the count values over a multinomial distribution by using 128 Monte Carlo samples to estimate the Dirichlet distribution for each sample (Fernandes et al., 2013). The Dirichlet modeling and iqlr transformation enabled us to perform valid significance tests among samples of different groups for DE analysis. This approach has been shown to be consistent with (but more conservative, i.e., fewer false positives, than) those of traditional DE analyses (Quinn, Crowley, & Richardson, 2018a).

### 2.5 Technical and diagnostic performance

Because the samples from intermediate areas were sequenced on a different run of the sequencing machine than the core and front samples, we needed to rule out a batch effect, in which samples from intermediate areas may have had disproportionately higher or lower numbers of reads for each transcript due to technical variation in sequencing performance during different runs. This could result in erroneous DE calls. Thus, we used the previously added ERCC control mixes (Ambion) to assess whether or not there was a batch effect due to sequencing run. Each set contains four groups of sequences with different ratios between the two mixes, representing “known” differences in abundance (mix 1 vs. mix 2 fold changes: 4:1, 1:1, 1:1.5, 1:2). We used the R (Team, 2016) package erccdashboard v1.10.0 (Munro et al., 2014) to analyze the counts of these sequences and generate receiver operator characteristic (ROC) curves and the area under the curve (AUC) statistic, lower limit of DE detection estimates (LODR), and expression ratio variability and bias measures based on these sequence abundance ratios (Figure S1). In the ROC curves, the AUC for two sets of true‐positive ERCC sequences (4:1 and 1:2) was 1.0, indicating perfect diagnostic performance, and the AUC for the third (1:1.5) was 0.89, indicating good diagnostic performance (Figure S1b). The MA plot shows that the measured ratios in our ERCC sequences converge around the *r*_m_ corrected ratios, indicating low variability (Figure S1c). Finally, the LODR plot indicated that DE *p*‐values were lower for ERCC sequences with wider ratios (i.e., 4:1 has the lowest *p*‐values, then 1:2, then 1:1.5), which is expected because the most pronounced fold change differences should yield the highest DE significance (Figure S1d). From these results, we inferred that the observed relative abundances between mixes of each set of ERCC sequences were close to the known relative abundances, and thus, batch effects do not appear to have occurred.

We also examined the counts of the invariant (1:1) group across all samples. Seven invariant ERCC transcripts remained after count filtering (same as used for the DE testing); we generated a boxplot of their counts, normalized by library size (Figure S2). The consistency of the boxplot distributions of the seven invariant ERCC sequences further indicated that there was no batch effect. Because the erccdashboard package indicated that the sets of true‐positive ERCC sequences (4:1, 1:2, 1:1.5) existed in observed ratios close to the known ratios, and because the invariant sequences (1:1) exhibited consistency across samples, we proceeded with downstream analyses.

### 2.6 Differential gene expression in discrete phases of the invasion

After applying a log‐ratio transformation to the count data, we were able to implement statistical tests that would otherwise be invalid for relative data. We grouped populations by phase (Daintree and Gordonvale in QLD/the core, Cape Crawford and Timber Creek in NT/intermediate areas, and Durack River and Halls Creek in WA/the front) and used these as groups for DE analysis. We fitted our log‐transformed count data to a nonparametric generalized linear model (glm) in ALDEx2. We took a “one versus all” approach, in which we compared samples from each state to samples from the other two states collectively (e.g., core vs. intermediate + front, intermediate vs. core + front, front vs. core + intermediate) using the Kruskal–Wallis test. This test design allowed us to identify transcripts that were up‐ and downregulated in toads from each state relative to those throughout the rest of the range. We only retained transcripts with Benjamini–Hochberg (FDR) corrected *p* < 0.05 (Fernandes et al., 2013). We detected 1,151 differentially expressed transcripts across all samples. We calculated the effect sizes of the differences between groups for each transcript, with positive values indicating upregulation, and negative values indicating downregulation. We further investigated all transcripts with effect sizes >1.5 or <−1.5.

### 2.7 Spatial gene expression patterns across the range

The DE testing performed in ALDEx2 generates differences between discrete groups; however, our data are sampled across a continuous variable: space. So, to visualize expression patterns across the toad's Australian range, we performed soft (fuzzy c‐means) clustering on our log‐transformed count data (with samples grouped by collection site, and sites ordered from east to west) using the R package Mfuzz v2.34.0 (Kumar & Futschik, 2007). The fuzzy c‐means algorithm groups transcript together based on similar expression patterns (using a fuzzifier parameter, *m*) across conditions to identify prominent, recurring patterns (clusters). Each transcript within a cluster is assigned a membership value, indicating how closely its expression pattern aligns with that of the cluster to which it belongs. To prevent random data from being clustered together, we used the mestimate command in the Mfuzz package to determine the optimal fuzzifier parameter value using a relation proposed for fuzzy c‐means clustering (Schwämmle & Jensen, 2010). We then used the cselection and Dmin commands to determine the optimal number of clusters, *c*, to generate. The results of both tools suggested using four clusters (*c* = 4); however, these tools need to be used with caution because automatic determination of the optimal value of *c* is difficult, and it is advised to review the data before choosing (Kumar & Futschik, 2007). For this reason, we manually performed repeated clustering for a range of *c* (*c* = 3, 4, 5, 6, 7, 8) using the fuzzy c‐means algorithm to visualize the differences in clusters of expression patterns across space and to compare the internal cores (identities and membership values of transcripts within each cluster) across *c* values. Although internal cores were consistent across all *c* values, we determined that several uniquely shaped expression patterns were collapsed at *c* = 4, and that these expression patterns only became separate at *c* = 6. At *c* > 6, redundant patterns began to emerge. For this reason, we selected *c* = 6 as the final value with which to perform soft clustering. We required a minimum membership value of 0.7 for all transcripts to their respective clusters.

### 2.8 Environmentally influenced gene expression

Genes affected by natural selection may have expression levels that are associated with environmental variables. We downloaded climatic data from the BioClim database (Hijmans, Cameron, Parra, Jones, & Jarvis, 2005) using the raster package (Hijmans, 2015) in R. Because different areas of Australia vary in aridity, we downloaded data on rainfall during the driest quarter and maximum temperature in the warmest month; these data are averages of annual statistics over the period of 1970 to 2000. We then used the lfmm v2.0 package (Frichot & Francois, 2015) in R to perform a latent factor mixed model (LFMM) to test the association between the log‐transformed count values of every expressed transcript and these two environmental variables. We applied a Benjamini–Hochberg correction to all *p*‐values from the LFMM.

### 2.9 Coordination in gene expression

To identify genes with coordinated (coassociated) expression, we calculated proportionality (ρ) between all pairs of transcripts in our dataset using the propr package (Quinn, Richardson, et al., 2017). A full description of this analysis is available in the Supplementary Information.

### 2.10 Annotation and gene ontology enrichment

We performed gene ontology (GO) enrichment analysis to identify the most enriched (recurring) biological functions in which our transcripts were involved. This allowed us to determine whether certain functional categories from the GO database were overrepresented in our DE, fuzzy clustering (spatial expression), and proportionality (coordination and differential coordination) datasets more than would be expected by chance. We used the Bioconductor tool (Huber et al., 2015) GOseq v 1.26.0 (Young, Wakefield, Smyth, & Oshlack, 2010) because it accounts for bias introduced by variation in transcript lengths. We assessed three sets of GO categories (Biological Process, Molecular Function, and Cellular Component) for enrichment using the *Wallenius approximation* (while controlling for transcript length) to test for over‐representation, and then Benjamini–Hochberg corrected the resulting *p*‐values. To visualize the results, we plotted significantly enriched GO categories with REVIGO (Supek, Bosnjak, Skunca, & Smuc, 2011), which performs SimRel (Sæbø, Almøy, & Helland, 2015) semantic clustering of similar GO functions with annotations sourced from the UniProt database (Consortium T.U., 2017). We then used GO terms to filter through the output lists from our DE, fuzzy clustering, and proportionality datasets to identify transcripts of genes involved in immune function.

### 2.11 Identification of immune genes

To identify additional transcripts that have known functions in the immune system (outside of those with the largest DE effect size or cluster membership), we cross‐matched the output transcript lists from all of our analyses with several lists of known immune genes within the database InnateDB (Breuer et al., 2013): Immunology Database and Analysis Portal (ImmPort), the Immunogenetic Related Information Source (IRIS), the MAPK/NFKB Network, and the Immunome Database. These databases consist entirely of human–mouse–bovine genes, but the immune systems of mammals and amphibians are broadly similar (Colombo, Scalvenzi, Benlamara, & Pollet, 2015; Robert & Ohta, 2009). We further investigated all transcripts within our datasets that matched a gene within the InnateDB gene lists.

### 2.12 Isolation by distance

To assess the effect of geographic distance on divergence in gene expression (thereby testing for isolation by distance), we performed a Mantel test using the ade4 package (Thioulouse & Dray, 2007). A full description of this analysis is available in the Appendix.

## 3 RESULTS

### 3.1 Identification of differentially expressed genes and their expression patterns

**Figure 2:**
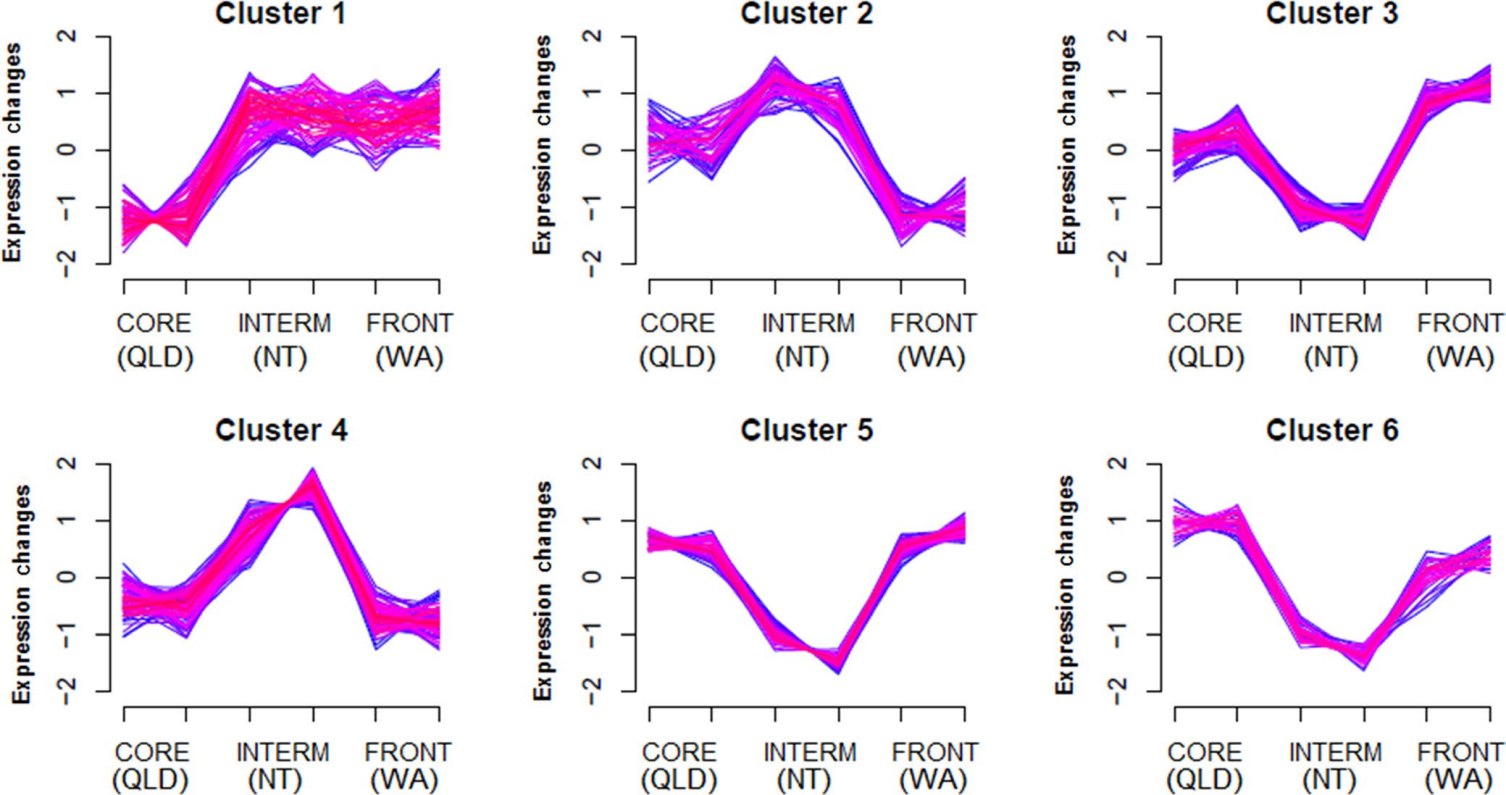
Six unique patterns of gene expression in spleen tissue from invasive cane toads (*Rhinella marina*). We collected samples in populations from areas spanning the invaded range in Australia (QLD = Queensland, NT = Northern Territory, WA = Western Australia). Color indicates membership values of genes to clusters (purple = 0.7–0.8; pink = 0.8–0.9; red = 0.9–1). Tick marks on the x‐axis indicate sites across the toad's Australian range in which spleens were collected (Figure 1)

**Figure 3:**
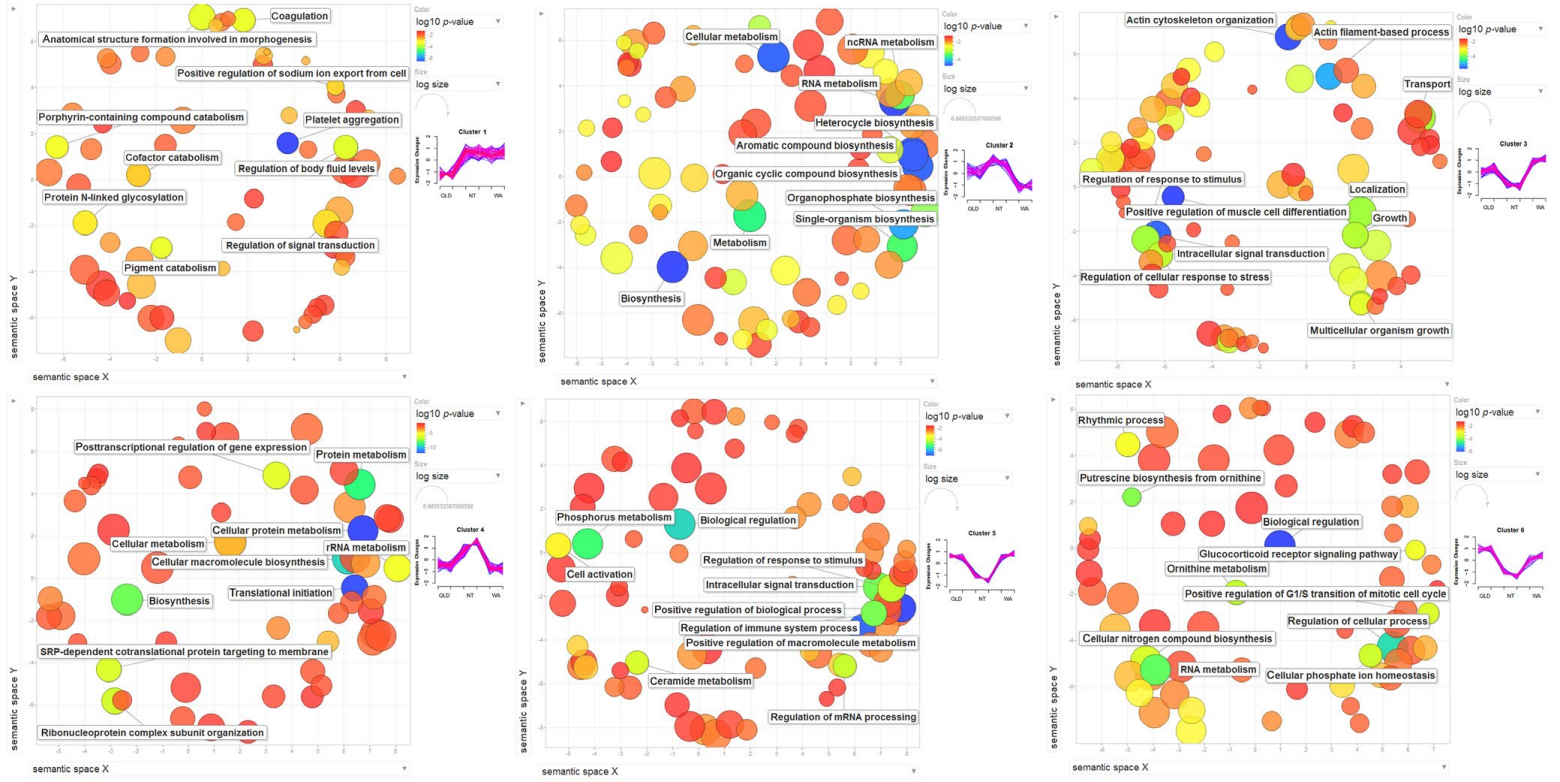
REVIGO plots displaying gene ontology (GO) terms (concepts/classes used to characterize gene function) depicting biological processes associated with transcripts following six major expression patterns in cane toads (*Rhinella marina*) across their Australian range (Figure 1). RNA‐Seq data from spleens were used to identify differentially expressed transcripts between invasion phases, then soft clustering was performed to visualize the expression patterns that these transcripts follow (Figure 2). Circles represent GO terms; those with the highest statistical significance are labeled. Circle size relates to breadth of GO terms. Colors show log_10_ *p*‐values

Overall, our DE analysis revealed 1,151 transcripts that were differentially expressed between invasion phases. These consisted of 131 transcripts with unique regulation in toads from the range core, 904 transcripts with unique regulation in toads from intermediate areas, and 122 transcripts with unique regulation in toads from the invasion front (list of transcripts in each phase in Appendix S1). Soft clustering analysis revealed six prominent expression patterns (clusters) of differentially expressed genes across the invasion (Figure 2): these clusters correspond to up‐ and downregulation of each invasion phase (core, intermediate, front). Only 340 of the 1,151 differentially expressed transcripts had sufficiently high membership values to fit within these six clusters (list of transcripts in each cluster in Appendix S1). The first cluster depicts low expression at the core and equally high expression throughout the rest of the range. The other five clusters all depict curvilinear patterns (in which expression is either highest or lowest in toads from intermediate areas), but vary in the expression levels in toads on the ends of the range. Gene ontology enrichment of each cluster from Figure 2 is shown in Figure 3, and functional characterization of each cluster based on individual transcript investigation is shown in Table 1.

**Table 1:** Most common functions of transcripts in each of the six major expression patterns in cane toads (*Rhinella marina*) across their Australian range (Figure 1). RNA‐Seq data from spleens were used to identify differentially expressed transcripts between invasion phases; then, soft clustering was performed to visualize the expression patterns that these transcripts follow (Figure 2)

Cluster	Number of transcripts	Most common biological function	Most significantly enriched GO term(s)	Less common biological functions
1	71	Blood coagulation/circulation (24 transcripts)	Platelet aggregation	Signal transduction, immune function, transcription regulation, viral processes
2	49	None	Cellular metabolism, biosynthesis	Metabolism, biosynthesis, cell cycle regulation, protein ubiquitination, translation initiation, transcription regulation
3	66	Signal transduction (24 transcripts)	Intracellular signal transduction, actin cytoskeleton organization	Protein transport, immune function, smooth muscle contraction, angiogenesis, cell cycle regulation
4	75	Translation initiation (28 transcripts)	Translation initiation	Metabolism, transcription regulation, protein ubiquitination, cell cycle regulation
5	49	Immune function (12 transcripts)	Regulation of biological process	Transcription regulation, signal transduction, cell cycle regulation, metabolism
6	30	None	Biological regulation	Transcription regulation, signal transduction, cell cycle regulation, immune function

### 3.2 Immune genes

The most common immune functions that we found among our differentially expressed transcripts were activation (list of transcripts and their expression patterns/clusters in Table 2a) and suppression (Table 2b) of inflammatory pathways, and cytotoxicity (Table 2c). Immune genes with other roles were too few to allow clear inferences to be drawn. Most proinflammatory transcripts were downregulated in toads from intermediate areas relative to toads from the range core and invasion front. This trend is reflected in the spatial expression data; in the fifth cluster (low expression in intermediate areas, high expression on either end of the range), approximately one‐quarter of transcripts were identified as relating to immune function (the highest proportion of any cluster), and this is the only cluster in which a GO term directly related to immunity was among the most significant (Figure 3, Table 1). Furthermore, additional immune transcripts were seen in the third and sixth clusters (which also exhibit lowest expression in toads from intermediate areas). Conversely, in the fourth cluster (high expression in intermediate areas, low expression on either end of the range), no immune transcripts were identified and all the most significant GO terms are related to translation (Figure 3, Table 1). Nonetheless, a few pro‐ and anti‐inflammatory transcripts were upregulated in intermediate toads (Table 2).

**Table 2:** Genes involved in immune function that are differentially expressed across the range of Australian cane toads. Spleens were collected from toads in the range core (QLD: Gordonvale and Daintree, *N* = 5 each), intermediate areas (NT: Cape Crawford and Timber Creek, *N* = 4 each), and invasion front (WA: Caroline Pool and Durack River, *N* = 5 each). Soft clustering was performed to visualize differential expression patterns between different phases of the invasion (Figure 2)

Gene	Protein	Expression pattern
(a) Inflammation		
*MAP3K2*	Mitogen‐activated protein kinase kinase kinase 2	NT down; Cluster 5
*pik3r5*	Phosphoinositide 3‐kinase regulatory subunit 5	NT down; Cluster 5
*CSF2RB*	Cytokine receptor common subunit beta	NT down; Cluster 5
*Srf*	Serum response factor	NT down; Cluster 5
*PTK2B*	Protein‐tyrosine kinase 2‐beta	NT down; Cluster 5
*PYCARD* (isoform 1)	Apoptosis‐associated speck‐like protein containing a CARD (ASC)	NT down; Cluster 5
*PYCARD* (isoform 2)	Apoptosis‐associated speck‐like protein containing a CARD (ASC)	NT down; Cluster 6
*Nlrp1b*	NACHT; LRR and PYD domains‐containing protein 1b allele 3 (NLRP1b)	NT down; Cluster 6
*ANKRD17*	Ankyrin repeat domain‐containing protein 17	NT down; Cluster 6
*mapk8*	Mitogen‐activated protein kinase 8	NT down; Cluster 6
*SPAG9*	C‐Jun‐amino‐terminal kinase‐interacting protein 4	NT down; Cluster 3
*Tab1*	TGF‐beta‐activated kinase 1 and MAP3K7‐binding protein 1	NT down; Cluster 3
*CAMK2G*	Calcium/calmodulin‐dependent protein kinase type II subunit gamma	NT down; Cluster 3
*TNFAIP2*	Tumor necrosis factor alpha‐induced protein 2	WA up; NT down; Cluster 3
*PYCARD* (isoform 3)	Apoptosis‐associated speck‐like protein containing a CARD (ASC)	NT down
*P2RX7*	P2X purinoceptor 7 (P2P7)	NT down
*NFATC2*	Nuclear factor of activated T‐cells; cytoplasmic 2	NT down
*Rps6ka3*	Ribosomal protein S6 kinase alpha−3	NT down
*PIK3CB*	Phosphatidylinositol 4;5‐bisphosphate 3‐kinase catalytic subunit beta isoform	NT down
*NOS2*	Nitric oxide synthase; inducible	NT down
*MAP4K5*	Mitogen‐activated protein kinase kinase kinase kinase 5	NT down
*HIPK1*	Homeodomain‐interacting protein kinase 1 (HIP1)	NT down
*STAT1*	Signal transducer and activator of transcription 1 (STAT1)	NT down
*Prkcb*	Protein kinase C beta type	NT down
*RIPK3*	Receptor‐interacting serine/threonine‐protein kinase 3 (RIPK3)	NT down
*Pak2*	Serine/threonine‐protein kinase PAK 2	NT down
*TRPC4AP*	Short transient receptor potential channel 4‐associated protein	NT down
*Nsmaf*	Protein FAN	NT down
*PLEKHG5*	Pleckstrin homology domain‐containing family G member 5	NT down
*IL7R*	Interleukin−7 receptor subunit alpha	NT down
*Tnfrsf21*	Tumor necrosis factor receptor superfamily member 21	NT down
*Rxra*	Retinoic acid receptor RXR‐alpha	NT down
*TRAF2*	TNF receptor‐associated factor 2	NT down
*IKBKE*	Inhibitor of nuclear factor kappa‐B kinase subunit epsilon	NT down
*Map3k14*	Mitogen‐activated protein kinase kinase kinase 14	NT down
*MTOR*	Serine/threonine‐protein kinase mTOR	NT down
*gtpbp1*	GTP‐binding protein 1	NT down
*ADAMTS1*	A disintegrin and metalloproteinase with thrombospondin motifs 1	NT down
*Erc1*	ELKS/Rab6‐interacting/CAST family member 1	NT down
*ARHGEF17*	Rho guanine nucleotide exchange factor 17	NT down; WA up
*Dele*	Death ligand signal enhancer	WA up; Cluster 3
*Cd84*	SLAM family member 5	WA up
*F2RL2*	Proteinase‐activated receptor 3	WA up
*TNFRSF19*	Tumor necrosis factor receptor superfamily member 19	WA up
*IL4R*	Interleukin−4 receptor subunit alpha	WA up
*mapk1*	Mitogen‐activated protein kinase 1	WA up
*Irgc* (isoform 1)	Interferon‐inducible GTPase 5	QLD up
*Irgc* (isoform 2)	Interferon‐inducible GTPase 5	QLD up
*Irgc* (isoform 3)	Interferon‐inducible GTPase 5	QLD up
*Irgc* (isoform 4)	Interferon‐inducible GTPase 5	QLD up
*Irgc* (isoform 5)	Interferon‐inducible GTPase 5	QLD up
*mul1a*	Mitochondrial ubiquitin ligase activator of nfkb 1‐A	QLD down; Cluster 1
*CSF2RA*	Granulocyte‐macrophage colony‐stimulating factor receptor subunit alpha	QLD down; Cluster 1
*TRIM25*	E3 ubiquitin/ISG15 ligase TRIM25	QLD down
*PYCARD* (isoform 4)	Apoptosis‐associated speck‐like protein containing a CARD (ASC)	NT up
*ecsit*	Evolutionarily conserved signaling intermediate in Toll pathway; mitochondrial	NT up
*NKAP*	NF‐kappa‐B‐activating protein	NT up
(b) Anti‐Inflammation		
*Tank*	TRAF family member‐associated NF‐kappa‐B activator	NT down; Cluster 5
*ERBIN*	Erbin	NT down; Cluster 5
*Itch*	E3 ubiquitin‐protein ligase Itchy	NT down; Cluster 5
*Sbno1*	Protein strawberry notch homolog 1	NT down; Cluster 5
*Smad6*	Mothers against decapentaplegic homolog 6	NT down; Cluster 3
*inpp5d*	Phosphatidylinositol 3,4,5‐trisphosphate 5‐phosphatase 1	NT down
*SBNO2*	Protein strawberry notch homolog 2	NT down
*SYNCRIP*	Heterogeneous nuclear ribonucleoprotein Q	NT down
*ATF3*	Cyclic AMP‐dependent transcription factor ATF−3	NT down
*Dusp4*	Dual specificity protein phosphatase 4	NT down
*Rps6ka4*	Ribosomal protein S6 kinase alpha−4	NT down
*AHR*	Aryl hydrocarbon receptor	NT down
*PTPRE*	Receptor‐type tyrosine‐protein phosphatase epsilon	NT down
*HAX1*	HCLS1‐associated protein X−1	NT up
*ppp4c*	Serine/threonine‐protein phosphatase 4 catalytic subunit	NT up
*Nlrc3* (isoform 1)	Protein NLRC3	NT up
*Nlrc3* (isoform 2)	Protein NLRC3	NT up
*ANXA1*	Annexin A1	NT up
*ciapin1*	Anamorsin	NT up
*impdh2*	Inosine−5'‐monophosphate dehydrogenase 2	NT up
*DHCR24*	Delta(24)‐sterol reductase	NT up
*CD200R1B*	Cell surface glycoprotein CD200 receptor 1‐B	QLD down; Cluster 1
*TNFRSF6B*	Tumor necrosis factor receptor superfamily member 6B	QLD down
(c) Cytotoxicity		
*STXBP2*	Syntaxin‐binding protein 2	NT down; Cluster 5
*NCR3LG1* (isoform 1)	Natural cytotoxicity triggering receptor 3 ligand 1	NT down; Cluster 5
*NCR3LG1* (isoform 2)	Natural cytotoxicity triggering receptor 3 ligand 1	NT down
*NCR3LG1* (isoform 3)	Natural cytotoxicity triggering receptor 3 ligand 1	NT down
*NCR3LG1* (isoform 4)	Natural cytotoxicity triggering receptor 3 ligand 1	NT down
*Slamf7*	SLAM family member 7	NT down; WA up

### 3.3 Climate‐influenced gene expression

Our LFMM revealed eleven transcripts with expression levels associated with maximum temperature during the hottest month, rainfall during the driest quarter, or both (list of transcripts in Appendix S2). Three of these transcripts followed the expression pattern of the first cluster (low expression at the range core, high expression throughout the rest of the range): Two are involved in cell adhesion and platelet activity, and function of the third is unknown. Two other transcripts were also downregulated at the core (but not a member of the first cluster); these are involved in transcription regulation and metabolism. Conversely, two transcripts involved in inflammation activation were upregulated at the core. Another transcript, involved in cell cycle regulation, was upregulated in intermediate areas. The three remaining transcripts were not differentially expressed: The first is involved in cell signaling in response to damage, the second is involved in blood circulation and response to nitric oxide (NO), and the third is unknown.

### 3.4 Coordination in gene expression

We tested whether some transcripts were coassociated across invasion phases by examining the expected value of ρ metric (Quinn, Richardson, et al., 2017), but only found large groups of coassociated transcripts involved in fundamental cellular processes such as translation (Figure S3; list of proportional transcripts in Appendix S3).

### 3.5 Isolation by distance

**Figure 4:**
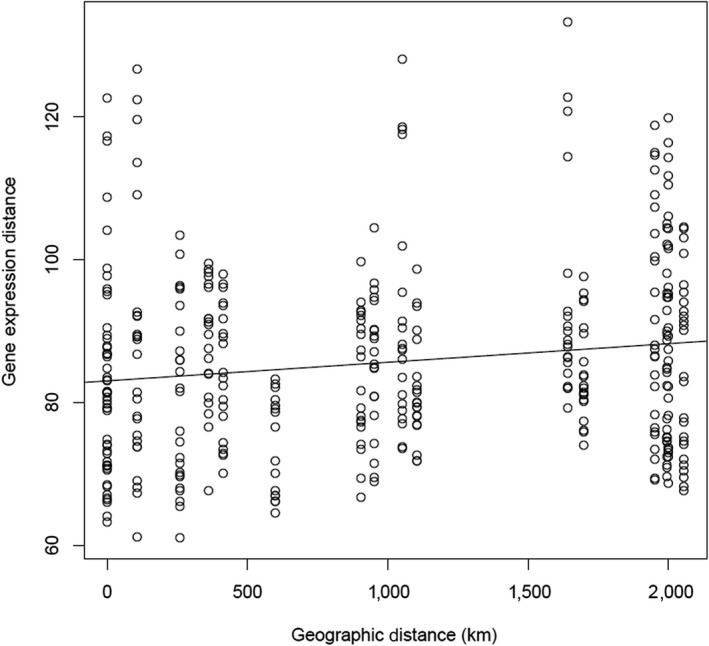
Correlation between geographic distance and gene expression distance of invasive cane toad (*Rhinella marina*) populations across their Australian range (Figure 1). Euclidean distances in geographic location and gene expression between populations were calculated using the dist function in R. A mantel test (performed with the ade4 package) confirmed that these were significantly correlated (*p* = 0.005)

Our Mantel test revealed a significant relationship between geographic distance and gene expression distance (*p* = 0.005, *R* ^2^ = 0.02; Figure 4).

## 4 DISCUSSION

The expression patterns of our differentially expressed genes suggest that multiple evolutionary forces may be at work. The curvilinearity in expression of many of our differentially expressed genes resembles that of other phenotypic traits affected by spatial sorting, such as leg length (Hudson et al., 2016). Physical activity can have transgenerational effects on gene expression (Barres & Zierath, 2016; Murashov et al., 2016), so dispersal may directly affect expression patterns. Additionally, the significant result of our Mantel test could be due to either genetic drift or a balance between geographically varying selection and gene flow (Endler, 1977). Although this result may be influenced by environmental gradients, it is unlikely that selection driven by environmental factors would act on a genome‐wide level (all expressed transcripts were used in our calculations for gene expression distance). This result suggests that genetic drift may be responsible for isolation by distance in Australian cane toads. Because spatial sorting and genetic drift drive nonadaptive variation, their effects may obscure adaptive variation, particularly when they act on the same traits as selection (i.e., physical activity is also linked to expression of inflammatory genes; Baynard, Vieira‐Potter, Valentine, & Woods, 2012; Gjevestad, Holven, & Ulven, 2015).

We also searched for evidence of adaptation to environment across the Australian invasive range, which is more similar to the native range in the east of Australia, and warmer and drier in the west. The expression pattern depicted in our first cluster (low expression in the core, high throughout the rest of the range) matches the trend across the Australian range in temperature and is opposite to the trend in rainfall. Population genetic structure in toads may be driven by these environmental variables (Selechnik et al., 2019). Three of the eleven transcripts with expression levels associated with temperature or rainfall belonged to this first cluster; the rest were not members of any of the six clusters. Two out of these three are involved in blood clotting, as are most of the transcripts in the first cluster. Proportionality analysis revealed two small groups of clotting‐related transcripts with coordinated expression. Blood clotting is affected by hydration levels (El‐Sabban, Fahim, Al Homsi, & Singh, 1996), and excessive blood clotting can impair health (PubmedHealth, 2014). Changes to the rate of blood clotting may reflect hydric‐related adaptations of intermediate and frontal toads living in drier conditions. Furthermore, four candidate SNP loci (with outlier *F*_ST_ values and associations with rainfall, indicating they may be under selection) have previously been found in a gene involved in blood clotting (Selechnik et al., 2019). However, if lowering blood clotting rates is indeed an adaptation to aridity in toads from intermediate areas and the front, then one might expect that transcripts promoting blood clotting would be downregulated in these individuals (yet in our study, they are upregulated). This may be because toads were collected from the wild and the environment was not controlled for. A common‐garden experiment with toads from across the range may reveal whether modification of blooding clotting rates is an evolved adaptation to aridity or simply a physiological reaction to different environments. Nonetheless, the differences in the expression of genes underlying these traits supports the hypothesis that cane toads are adapting to their abiotic environment.

The conceptual scheme of Lee and Klasing (2004), based on the ERH, predicts that the expression of genes encoding mediators of costly immune responses (e.g., inflammation) would follow a density‐driven curvilinear pattern, with highest expression in toads from intermediate areas (where density is presumably highest, facilitating parasite transmission). However, most immune transcripts (involved pro‐ or anti‐inflammatory signaling) in our study followed the opposite pattern: curvilinear, but with lowest expression in toads from intermediate areas. Although not all immune transcripts followed this pattern, our data largely do not support the enemy release hypothesis. It must be noted that our inferences are based on gene annotations sourced from many different taxa. These genes may not function the same way in cane toads as they do in the organisms from which they were described. Furthermore, although we only sampled toads that appeared to be healthy, the infection status of each toad was not assayed, manipulated nor controlled for. Thus, spatial heterogeneity in pathogen pressure or environmental conditions may play a role in gene expression; however, parasite prevalence is generally higher in intermediate‐age populations than in younger and older populations (Freeland et al., 1986), inconsistent with the general pattern for downregulation of immune transcripts in toads from intermediate‐age populations. Our results may be driven by other environmental variables or may have a genetic basis through polymorphism in promoter regions, which are not sequenced when using RNA‐Seq (Wang, Gerstein, & Snyder, 2009).

Our findings are consistent with previous phenotypic data on toads: Spleen sizes and fat body masses also follow a curvilinear pattern in which they are lowest in toads from intermediate parts of the range (Brown, Kelehear, et al., 2015b). If toads indeed follow a “traveling wave” density pattern, then the increased densities that occur several years postcolonization may not just heighten pathogen‐mediated selection, but also intraspecific competition; reduced access to food resources may thus constrain the toad's ability to increase investment in immunity (Brown, Kelehear, et al., 2015b). This may explain smaller spleen sizes and lower expression of transcripts involved in inflammatory immune responses in toads from intermediate areas. Availability of energetic resources was not considered in the predictions of Lee and Klasing (2004), and although individuals at an invasion front may have a reduced benefit from energetic investment into immunity, they may invest anyway due to reduced intraspecific competition for food. Thus, the strength of immune functioning in invasion front individuals may depend on factors other than enemy release, such as prey abundance and interspecific competition.

## 5 CONCLUSIONS

Although natural selection causes adaptive change in immune function in response to infection, this can co‐occur with selection by abiotic environmental factors, as well as nonadaptive variation driven by forces such as spatial sorting and genetic drift, particularly in invasive species. Because these forces can exert different effects on the same traits, predicting trait variation based solely on selection may not be an accurate approach. Nonetheless, methods such as RNA‐Seq remain a powerful tool for uncovering the diverse and sometimes opposing forces that underpin rapid evolution in invasive species. The expression patterns that we observed in pro‐ and anti‐inflammatory transcripts generally do not support the predictions of Lee and Klasing (2004) based on the ERH. Notably, our study suggests that the predicted consequences of enemy release are difficult to study in the wild, especially in the absence of infection data, because host–parasite associations are affected by many factors. Common‐garden studies could be used to test these alternative hypotheses, but it is worth noting that keeping animals in captivity may also have functional impacts, sometimes making it difficult to interpret these results in the context of wild populations. These complicated dynamics are likely to explain why support for the predictions of ecological hypotheses, including ERH, is often inconsistent.

## CONFLICT OF INTEREST

None declared.

## AUTHOR CONTRIBUTIONS

Dan Selechnik, Mark Richardson, Lee Ann Rollins, Greg Brown, and Richard Shine designed the experiment. Dan Selechnik, Mark Richardson, and Lee Ann Rollins performed data collection. Dan Selechnik, Mark Richardson, and Lee Ann Rollins performed data analysis. Dan Selechnik wrote the manuscript. Mark Richardson, Lee Ann Rollins, Greg Brown, and Richard Shine revised the manuscript.

## References

[ece35249-bib-0001] Aitchison, J. , & Egozcue, J. J. (2005). Compositional data analysis: Where are we and where should we be heading? Mathematical Geology, 37, 829–850. 10.1007/s11004-005-7383-7

[ece35249-bib-0002] Andrews, S. (2010). FastQC: A quality control tool for high throughput sequence data. Retrieved from http://www.bioinformatics.babraham.ac.uk/projects/fastqc

[ece35249-bib-0003] Barres, R. , & Zierath, J. R. (2016). The role of diet and exercise in the transgenerational epigenetic landscape of T2DM. Nature Reviews Endocrinology, 12, 441–451. 10.1038/nrendo.2016.87 27312865

[ece35249-bib-0004] Bax, N. , Williamson, A. , Aguero, M. , Gonzalez, E. , & Geeves, W. (2003). Marine invasive alien species: A threat to global biodiversity. Marine Policy, 27, 313–323. 10.1016/S0308-597X(03)00041-1

[ece35249-bib-0005] Baynard, T. , Vieira‐Potter, V. J. , Valentine, R. J. , & Woods, J. A. (2012). Exercise training effects on inflammatory gene expression in white adipose tissue of young mice. Mediators of Inflammation, 2012, 767953 10.1155/2012/767953 23319832 PMC3540918

[ece35249-bib-0006] Berthouly‐Salazar, C. , van Rensburg, B. J. , Le Roux, J. J. , van Vuuren, B. J. , & Hui, C. (2012). Spatial sorting drives morphological variation in the invasive bird, *Acridotheris tristis* PLoS ONE, 7, e38145 10.1371/journal.pone.0038145 22693591 PMC3364963

[ece35249-bib-0007] Bolger, A. M. , Lohse, M. , & Usadel, B. (2014). Trimmomatic: A flexible trimmer for Illumina sequence data. Bioinformatics, 30, 2114–2120. 10.1093/bioinformatics/btu170 24695404 PMC4103590

[ece35249-bib-0008] Breuer, K. , Foroushani, A. K. , Laird, M. R. , Chen, C. , Sribnaia, A. , Lo, R. , … Lynn, D. J. (2013). InnateDB: Systems biology of innate immunity and beyond—recent updates and continuing curation. Nucleic Acids Research, 41, D1228–D1233. 10.1093/nar/gks1147 23180781 PMC3531080

[ece35249-bib-0009] Brown, G. P. , Kelehear, C. , Shilton, C. M. , Phillips, B. L. , & Shine, R. (2015b). Stress and immunity at the invasion front: A comparison across cane toad (*Rhinella marina*) populations. Biological Journal of the Linnean Society, 116, 748–760.

[ece35249-bib-0010] Brown, G. P. , Kelehear, C. , & Shine, R. (2013). The early toad gets the worm: Cane toads at an invasion front benefit from higher prey availability. Journal of Animal Ecology, 82, 854–862. 10.1111/1365-2656.12048 23360501

[ece35249-bib-0011] Brown, G. P. , Phillips, B. L. , Dubey, S. , & Shine, R. (2015c). Invader immunology: Invasion history alters immune system function in cane toads (*Rhinella marina*) in tropical Australia. Ecology Letters, 18, 57–65. 25399668 10.1111/ele.12390

[ece35249-bib-0012] Brown, G. P. , Shilton, C. , Phillips, B. L. , & Shine, R. (2007) Invasion, stress, and spinal arthritis in cane toads. Proceedings of the National Academy of Sciences of the United States of America, 104, 17698–17700. 17951431 10.1073/pnas.0705057104 PMC2077021

[ece35249-bib-0013] Brown, G. P. , & Shine, R. (2014). Immune response varies with rate of dispersal in invasive cane toads (*Rhinella marina*). PLoS ONE, 9, 6708–11. 10.1371/journal.pone.0099734 PMC4061023 24936876

[ece35249-bib-0014] Bureau of Meteorology A.G. (2018) Climate data online. Commonwealth of Australia. Retrieved from http://www.bom.gov.au/

[ece35249-bib-0015] Clavero, M. , Brotonsa, L. , Pons, P. , & Sol, D. (2009). Prominent role of invasive species in avian biodiversity loss. Biological Conservation, 142, 2043–2049. 10.1016/j.biocon.2009.03.034

[ece35249-bib-0016] Colautti, R. I. , Ricciardi, A. , Grigorovich, I. A. , & MacIsaac, H. J. (2004). Is invasion success explained by the enemy release hypothesis? Ecology Letters, 7, 721–733. 10.1111/j.1461-0248.2004.00616.x

[ece35249-bib-0017] Colombo, B. M. , Scalvenzi, T. , Benlamara, S. , & Pollet, N. (2015). Microbiota and mucosal immunity in amphibians. Frontiers in Immunology, 6, 111 10.3389/fimmu.2015.00111 25821449 PMC4358222

[ece35249-bib-0018] Consortium T.U. (2017). UniProt: The universal protein knowledgebase. Nucleic Acids Research, 45, D158–D169. 27899622 10.1093/nar/gkw1099 PMC5210571

[ece35249-bib-0019] Cornet, S. , Brouat, C. , Diagne, C. , & Charbonnel, N. (2016). Eco‐immunology and bioinvasion: Revisiting the evolution of increased competitive ability hypotheses. Evolutionary Applications, 9, 952–962. 10.1111/eva.12406 27606004 PMC4999526

[ece35249-bib-0020] El‐Sabban, F. , Fahim, M. A. , Al Homsi, M. F. , & Singh, S. (1996). Dehydration accelerates in vivo platelet aggregation in pial arterioles of lead‐treated mice. Journal of Thermal Biology, 20, 469–476.

[ece35249-bib-0021] Endler, J. A. (1977) Geographic variation, speciation, and clines In Monographs in population biology (pp. 246). Princeton, NJ: Princeton University Press, University of Minnesota. 409931

[ece35249-bib-0022] Erb, I. , & Notredame, C. (2016). How should we measure proportionality on relative gene expression data? Theory in Biosciences, 135, 21–36. 10.1007/s12064-015-0220-8 26762323 PMC4870310

[ece35249-bib-0023] Fernandes, A. D. , Macklaim, J. M. , Linn, T. G. , Reid, G. , & Gloor, G. B. (2013). ANOVA‐like differential expression (ALDEx) analysis for mixed population RNA‐Seq. PLoS ONE, 8, e67019 10.1371/journal.pone.0067019 23843979 PMC3699591

[ece35249-bib-0024] Fernandes, A. D. , Reid, J. N. S. , Macklaim, J. M. , McMurrough, T. A. , Edgell, D. R. , & Gloor, G. B. (2014). Unifying the analysis of high‐throughput sequencing datasets: Characterizing RNA‐seq, 16S rRNA gene sequencing and selective growth experiments by compositional data analysis. Microbiome, 2, 15 10.1186/2049-2618-2-15 24910773 PMC4030730

[ece35249-bib-0025] Finnerty, P. , Shine, R. , & Brown, G. P. (2017) The costs of parasite infection: effects of removing lungworms on performance, growth and survival of free‐ranging cane toads. Functional Ecology, 32(2), 402–415.

[ece35249-bib-0026] Freeland, W. J. , Delvinquier, B. L. J. , & Bonnin, B. (1986). Food and parasitism of the cane toad, *Bufo‐marinus*, in relation to time since colonization. Australian Wildlife Research, 13, 489–499.

[ece35249-bib-0027] Frichot, E. , & Francois, O. (2015). LEA: An R package for landscape and ecological association studies. Methods in Ecology and Evolution, 6, 925–929.

[ece35249-bib-0028] Gjevestad, G. O. , Holven, K. B. , & Ulven, S. M. (2015). Effects of exercise on gene expression of inflammatory markers in human peripheral blood cells: A systematic review. Current Cardiovascular Risk Reports, 9, 34 10.1007/s12170-015-0463-4 26005511 PMC4439514

[ece35249-bib-0029] Gruber, J. , Brown, G. , Whiting, M. J. , & Shine, R. (2017). Geographic divergence in dispersal‐related behaviour in cane toads from range‐front versus range‐core populations in Australia. Behavioral Ecology and Sociobiology, 71 10.1007/s00265-017-2266-8

[ece35249-bib-0030] Hijmans, R. J. (2015) raster: Geographic data analysis and modeling.

[ece35249-bib-0031] Hijmans, R. J. , Cameron, S. E. , Parra, J. L. , Jones, P. G. , & Jarvis, A. (2005). Very high resolution interpolated climate surfaces for global land areas. International Journal of Climatology, 25, 1965–1978. 10.1002/joc.1276

[ece35249-bib-0032] Hilker, F. M. , Lewis, M. A. , Seno, H. , Langlais, M. , & Malchow, H. (2005). Pathogens can slow down or reverse invasion fronts of their hosts. Biological Invasions, 7, 817–832. 10.1007/s10530-005-5215-9

[ece35249-bib-0033] Huber, W. , Carey, V. J. , Gentleman, R. , Anders, S. , Carlson, M. , Carvalho, B. S. , … Morgan, M. (2015). Orchestrating high‐throughput genomic analysis with Bioconductor. Nature Methods, 12(2), 115–121. 10.1038/nmeth.3252 25633503 PMC4509590

[ece35249-bib-0034] Hudson, C. M. , Brown, G. P. , & Shine, R. (2016). It is lonely at the front: Contrasting evolutionary trajectories in male and female invaders. Royal Society Open Science, 3, 160687 10.1098/rsos.160687 28083108 PMC5210690

[ece35249-bib-0035] Janeway, C. A. , Travers, P. , & Walport, M. (2001). Immunobiology: The immune system in health and disease In Toledo M., Bochicchio A., Acevedo-Quinones C., Zayetz E., Divakaran D., Mickey R. K. & Livitt S. (Eds.), Immunobiology (pp. 6708–855). New York, NY: Garland Science.

[ece35249-bib-0036] Kelehear, C. , Webb, J. K. , & Shine, R. (2009). Rhabdias pseudosphaerocephala infection in *Bufo marinus*: Lung nematodes reduce viability of metamorph cane toads. Parasitology, 136, 919–927. 10.1017/S0031182009006325 19523249

[ece35249-bib-0037] Klasing, K. C. , & Leshchinsky, T. V. (1999). Functions, costs, and benefits of the immune system during development and growth. International Ornithology Congress, Proceedings, 2817–2832.

[ece35249-bib-0038] Kosmala, G. K. , Brown, G. P. , Christian, K. A. , Hudson, C. M. , & Shine, R. (2018). The thermal dependency of locomotor performance evolves rapidly within an invasive species. Ecology and Evolution, 8, 4403–4408. 10.1002/ece3.3996 29760882 PMC5938468

[ece35249-bib-0039] Kumar, L. , & Futschik, M. (2007). Mfuzz: A software package for soft clustering of microarray data. Bioinformation, 2, 5–7. 10.6026/97320630002005 18084642 PMC2139991

[ece35249-bib-0040] Lee, K. A. , & Klasing, K. C. (2004). A role for immunology in invasion biology. Trends in Ecology & Evolution, 19, 523–529. 10.1016/j.tree.2004.07.012 16701317

[ece35249-bib-0041] Lovell, D. , Pawlowsky‐Glahn, V. , Egozcue, J. J. , Marguerat, S. , & Bahler, J. (2015). Proportionality: A valid alternative to correlation for relative data. PLOS Computational Biology, 11, e1004075 10.1371/journal.pcbi.1004075 25775355 PMC4361748

[ece35249-bib-0042] Lowe, W. H. , Muhlfeld, C. C. , & Allendorf, F. W. (2015). Spatial sorting promotes the spread of maladaptive hybridization. Trends in Ecology & Evolution, 30, 456–462. 10.1016/j.tree.2015.05.008 26122483

[ece35249-bib-0043] Mader, G. , Castro, L. , Bonatto, S. L. , & de Freitas, L. (2016). Multiple introductions and gene flow in subtropical South American populations of the fireweed, Senecio madagascariensis(Asteraceae). Genetics and Molecular Biology, 39, 135–144. 10.1590/1678-4685-GMB-2015-0167 27007907 PMC4807391

[ece35249-bib-0044] Martin, L. B. , Hopkins, W. A. , Mydlarz, L. D. , & Rohr, J. R. (2010). The effects of anthropogenic global changes on immune functions and disease resistance. Annals of the New York Academy of Sciences, 1195, 129–148. 10.1111/j.1749-6632.2010.05454.x 20536821

[ece35249-bib-0045] McKean, K. A. , & Lazzaro, B. (2011) Mechanisms of life history evolution In Flatt T., & Heyland A. (Eds.), The costs of immunity and the evolution of immunological defense mechanisms (pp. 504). Oxford, UK: OUP Oxford.

[ece35249-bib-0046] Munro, S. A. , Lund, S. P. , Pine, P. S. , Binder, H. , Clevert, D.‐A. , Conesa, A. , … Salit, M. (2014). Assessing technical performance in differential gene expression experiments with external spike‐in RNA control ratio mixtures. Nature Communications, 5, 5125 10.1038/ncomms6125 25254650

[ece35249-bib-0047] Murashov, A. K. , Pak, E. S. , Koury, M. , Ajmera, A. , Jeyakumar, M. , Parker, M. , … Neufer, P. D. (2016). Paternal long‐term exercise programs offspring for low energy expenditure and increased risk for obesity in mice. The FASEB Journal, 30, 775–784. 10.1096/fj.15-274274 26506979 PMC4714554

[ece35249-bib-0048] Patro, R. , Duggal, G. , Love, M. I. , Irizarry, R. A. , & Kingsford, C. (2017). Salmon provides fast and bias‐aware quantification of transcript expression. Nature Methods, 14(4), 417–419. 10.1038/nmeth.4197 28263959 PMC5600148

[ece35249-bib-0049] Phillips, B. L. , Kelehear, C. , Pizzatto, L. , Brown, G. P. , Barton, D. I. , & Shine, R. (2010). Parasites and pathogens lag behind their host during periods of host range advance. Ecology, 91, 872–881. 10.1890/09-0530.1 20426344

[ece35249-bib-0050] PubmedHealth (2014). Excessive blood clotting. NIH. Retrieved from https://www.ncbi.nlm.nih.gov/pubmedhealth/PMH0062998/

[ece35249-bib-0051] Quinn, T. , Crowley, T. , & Richardson, M. F. (2018a). Benchmarking differential expression analysis tools for RNA‐Seq: Normalization‐based vs. log‐ratio transformation‐based methods. BMC Bioinformatics, 19, 274 10.1186/s12859-018-2261-8 30021534 PMC6052553

[ece35249-bib-0052] Quinn, T. , Erb, I. , Gloor, G. , Notredame, C. , Richardson, M. F. , & Crowley, T. M. (2018b). A field guide for the compositional analysis of any‐omics data. bioRxiv. 10.1101/484766 PMC6755255 31544212

[ece35249-bib-0053] Quinn, T. , Erb, I. , Richardson, M. F. , & Crowley, T. (2018c). Understanding sequencing data as compositions: an outlook and review. Bioinformatics, 34(16), 2870–2878. 10.1093/bioinformatics/bty175. 29608657 PMC6084572

[ece35249-bib-0054] Quinn, T. , Richardson, M. F. , Lovell, D. , & Crowley, T. (2017). propr: An R‐package for identifying proportionally abundant features using compositional data analysis. Scientific Reports, 7, 16252 10.1038/s41598-017-16520-0 29176663 PMC5701231

[ece35249-bib-0055] Richardson, M. F. , Sequeira, F. , Selechnik, D. , Carneiro, M. , Vallinoto, M. , Reid, J. G. , … Rollins, L. A. (2018). Improving amphibian genomic resources: A multi‐tissue reference transcriptome of an iconic invader. GigaScience, 7, 6708–7. 10.1093/gigascience/gix114 PMC5765561 29186423

[ece35249-bib-0056] Robert, J. , & Ohta, Y. (2009). Comparative and developmental study of the immune system in Xenopus. Developmental Dynamics, 238, 1249–1270. 19253402 10.1002/dvdy.21891 PMC2892269

[ece35249-bib-0057] Rollins, L. A. , Woolnough, A. P. , Wilton, A. N. , Sinclair, R. , & Sherwin, W. B. (2009). Invasive species can't cover their tracks: Using microsatellites to assist management of starling (*Sturnus vulgaris*) populations in Western Australia. Molecular Ecology, 18, 1560–1573. 19317845 10.1111/j.1365-294X.2009.04132.x

[ece35249-bib-0058] Russo, A. G. , Eden, J. S. , Enosi, T. D. , Shi, M. , Selechnik, D. , Shine, R. , … White, P. A. (2018). Viral discovery in the invasive Australian cane toad (*Rhinella marina*) using metatranscriptomic and genomic approaches. Journal of Virology, 92(17), e00768-18. 29899109 10.1128/JVI.00768-18 PMC6096826

[ece35249-bib-0059] Sæbø, S. , Almøy, T. , & Helland, I. S. (2015). simrel — A versatile tool for linear model data simulation based on the concept of a relevant subspace and relevant predictors. Chemometrics and Intelligent Laboratory Systems, 146, 128–135. 10.1016/j.chemolab.2015.05.012

[ece35249-bib-0060] Schwämmle, V. , & Jensen, O. N. (2010). A simple and fast method to determine the parameters for fuzzy c–means cluster analysis. Bioinformatics, 26, 2841–2848. 10.1093/bioinformatics/btq534 20880957

[ece35249-bib-0061] Selechnik D. , Richardson M. F. , Shine R. , Devore J. , Ducatez S. , & Rollins L. A. (2019). Bottleneck revisited: increased adaptive variation despite reduced overall genetic diversity in a rapidly adapting invader. bioRxiv, 10.1101/557868 PMC6901984 31850072

[ece35249-bib-0062] Selechnik, D. , Rollins, L. A. , Brown, G. P. , Kelehear, C. , & Shine, R. (2017a). The things they carried: The pathogenic effects of old and new parasites following the intercontinental invasion of the Australian cane toad (*Rhinella marina*). International Journal for Parasitology: Parasites and Wildlife, 6, 375–385. 10.1016/j.ijppaw.2016.12.001 30951567 PMC5715224

[ece35249-bib-0063] Selechnik, D. , West, A. J. , Brown, G. P. , Fanson, K. V. , Addison, B. A. , Rollins, L. A. , & Shine, R. (2017b). Effects of invasion history on physiological responses to immune system activation in invasive Australian cane toads. PeerJ, 5, e3856 10.7717/peerj.3856 29018604 PMC5633027

[ece35249-bib-0064] Shine, R. , Brown, G. P. , & Phillips, B. L. (2011). An evolutionary process that assembles phenotypes through space rather than through time. Proceedings of the National Academy of Sciences of the United States of America, 108, 5708–5711. 10.1073/pnas.1018989108 21436040 PMC3078378

[ece35249-bib-0065] Simberloff, D. , & Gibbons, L. (2004). Now you see them, now you don't! – population crashes of established introduced species. Biological Invasions, 6, 161–172. 10.1023/B:BINV.0000022133.49752.46

[ece35249-bib-0066] Spurgin, L. G. , & Richardson, D. S. (2010). How pathogens drive genetic diversity: MHC, mechanisms and misunderstandings. Proceedings of the Royal Society B: Biological Sciences, 277, 979–988. 10.1098/rspb.2009.2084 PMC2842774 20071384

[ece35249-bib-0067] Supek, F. , Bosnjak, M. , Skunca, N. , & Smuc, T. (2011). REVIGO summarizes and visualizes long lists of gene ontology terms. PLoS ONE, 6, e21800 10.1371/journal.pone.0021800 21789182 PMC3138752

[ece35249-bib-0068] Team, R. C. (2016). R: A language and environment for statistical computing. Vienna, Austria: R Foundation for Statistical Computing.

[ece35249-bib-0069] Thioulouse, J. , & Dray, S. (2007). Interactive multivariate data analysis in R with the ade4 and ade4TkGUI packages. Journal of Statistical Software, 22, 6708–6721.

[ece35249-bib-0070] Tingley, R. , Ward‐Fear, G. , Schwarzkopf, L. , et al. (2017). New weapons in the toad toolkit: A review of methods to control and mitigate the biodiversity impacts of invasive cane toads (*Rhinella marina*). The Quarterly Review of Biology, 92, 123–149. 29562120 10.1086/692167

[ece35249-bib-0071] Wang, Z. , Gerstein, M. , & Snyder, M. (2009). RNA‐Seq: A revolutionary tool for transcriptomics. Nature Reviews Genetics, 10 10.1038/nrg2484 PMC2949280 19015660

[ece35249-bib-0072] Young, M. D. , Wakefield, M. J. , Smyth, G. K. , & Oshlack, A. (2010). Gene ontology analysis for RNA‐seq: Accounting for selection bias. Genome Biology, 11, R14 10.1186/gb-2010-11-2-r14 20132535 PMC2872874

